# The Association between the Differential Expression of lncRNA and Type 2 Diabetes Mellitus in People with Hypertriglyceridemia

**DOI:** 10.3390/ijms24054279

**Published:** 2023-02-21

**Authors:** Shoumeng Yan, Nan Yao, Xiaotong Li, Mengzi Sun, Yixue Yang, Weiwei Cui, Bo Li

**Affiliations:** 1 Department of Epidemiology and Biostatistics, School of Public Health, Jilin University, Changchun 130021, China; 2 School of Nursing, Jilin University, Changchun 130021, China; 3 Department of Nutrition and Food Hygiene, School of Public Health, Jilin University, Changchun 130021, China

**Keywords:** type 2 diabetes mellitus, lncRNA, hypertriglyceridemia, MIN6, ceRNA

## Abstract

Compared with diabetic patients with normal blood lipid, diabetic patients with dyslipidemia such as high triglycerides have a higher risk of clinical complications, and the disease is also more serious. For the subjects with hypertriglyceridemia, the lncRNAs affecting type 2 diabetes mellitus (T2DM) and the specific mechanisms remain unclear. Transcriptome sequencing was performed on peripheral blood samples of new-onset T2DM (six subjects) and normal blood control (six subjects) in hypertriglyceridemia patients using gene chip technology, and differentially expressed lncRNA profiles were constructed. Validated by the GEO database and RT-qPCR, lncRNA ENST00000462455.1 was selected. Subsequently, fluorescence in situ hybridization (FISH), real-time quantitative polymerase chain reaction (RT-qPCR), CCK-8 assay, flow cytometry, and enzyme-linked immunosorbent assay (ELISA) were used to observe the effect of ENST00000462455.1 on MIN6. When silencing the ENST00000462455.1 for MIN6 in high glucose and high fat, the relative cell survival rate and insulin secretion decreased, the apoptosis rate increased, and the expression of the transcription factors Ins1, Pdx-1, Glut2, FoxO1, and ETS1 that maintained the function and activity of pancreatic β cells decreased (*p* < 0.05). In addition, we found that ENST00000462455.1/miR-204-3p/CACNA1C could be the core regulatory axis by using bioinformatics methods. Therefore, ENST00000462455.1 was a potential biomarker for hypertriglyceridemia patients with T2DM.

## 1. Introduction

Diabetes has become the third leading chronic disease that seriously endangers human health. In 2021, there were about 537 million people with diabetes worldwide, and this number is projected to reach 643 million by 2030 and 783 million by 2045. The prevalence of diabetes is on the rise, and over 6.7 million people will die from diabetes-related causes [1]. Type 2 diabetes mellitus (T2DM) is an endocrine and metabolic disease caused by a combination of genetic and environmental factors and characterized by fasting and postprandial hyperglycemia, which account for more than 90% of diabetes [2]. Existing evidence indicates that people with T2DM have a 15% increase in all-cause mortality compared with people without diabetes [3].

Pancreatic β cells play an essential role in maintaining glucose homeostasis [4]. Glucose is a major physiological regulator for pancreatic β cells and can be metabolized via pancreatic β cells, thereby stimulating insulin secretion [5,6]. However, in chronic hyperglycemic environments and sustained glucose metabolism, pancreatic β cells are prone to damage and dysfunction, resulting in defective insulin secretion [7]. In addition, dyslipidemia also plays an important role in the development of T2DM. On the one hand, the lipotoxicity caused by dyslipidemia could affect the development of insulin resistance, which in turn aggravates the occurrence of lipid metabolism disorders, and a vicious circle is established [8]. On the other hand, the accumulation of abnormally elevated triglycerides in pancreatic β cells leads to their dysfunction and the further apoptosis of pancreatic β cells, which eventually causes the disorder of insulin secretion and the increase of blood glucose, thus inducing T2DM [9]. Meanwhile, T2DM complicated with hyperlipidemia is more likely to induce complications such as cardiovascular and cerebrovascular diseases [10]. Therefore, whether from a public health or a clinical perspective, hypertriglyceridemia patients with T2DM should be paid more attention.

Long noncoding RNAs (lncRNAs) represent a class of transcripts longer than 200 nucleotides with limited protein-coding potential [11]. They affect downstream gene expression and promote/inhibit disease development mainly by binding to targeted mRNAs or serving as endogenous competing RNAs for miRNAs [12]. Studies have found that lncRNAs are related to the development of T2DM and its related diseases. For example, lncRNA PVT1 can regulate insulin secretion and lipid metabolism by affecting miR-20a-5p expression, and it is also associated with end-stage renal disease in T2DM patients [13,14]. The lncRNA MALAT 1 plays an important role in the pathophysiology, inflammation, and progression of T2DM through regulating gene transcription [15]. MEG3 is overexpressed in patients with T2DM and is closely related to the occurrence of diabetic retinopathy [16]. Meanwhile, more than 1000 lncRNAs have been found in human islet cells, many of which are highly islet-specific, suggesting that they could have important and unique roles in regulating pancreatic function [13]. Our study aims to screen the differentially expressed lncRNA between new-onset T2DM and normal blood glucose control in hypertriglyceridemia subjects, and then explore the effects and possible mechanism of lncRNA on pancreatic β cell function and activity, thus providing some references for the prevention and treatment of T2DM in people with hypertriglyceridemia.

## 2. Results

### 2.1. Screening and Validation of Differentially Expressed lncRNAs

Blood samples of six newly diagnosed T2DM patients and six patients with normal blood glucose were used to perform RNA sequencing. Basic information of subjects and the situation of data filtering are shown in Tables S1 and S2, respectively. The cleaned data is used for subsequent analysis to ensure the quality of the analysis. We obtained a total of 3163 differentially expressed lncRNAs (1439 up and 1724 down) between the T2DM group and the control group based on a *p* value less than or equal to 0.05. The corresponding volcano plot and heat map are shown in Figure S1. Meanwhile, a total of 25 differentially expressed lncRNAs (10 up and 15 down) were found between the above two groups based on an adjusted *p* value less than or equal to 0.05 (Table 1).

**Figure 1:**
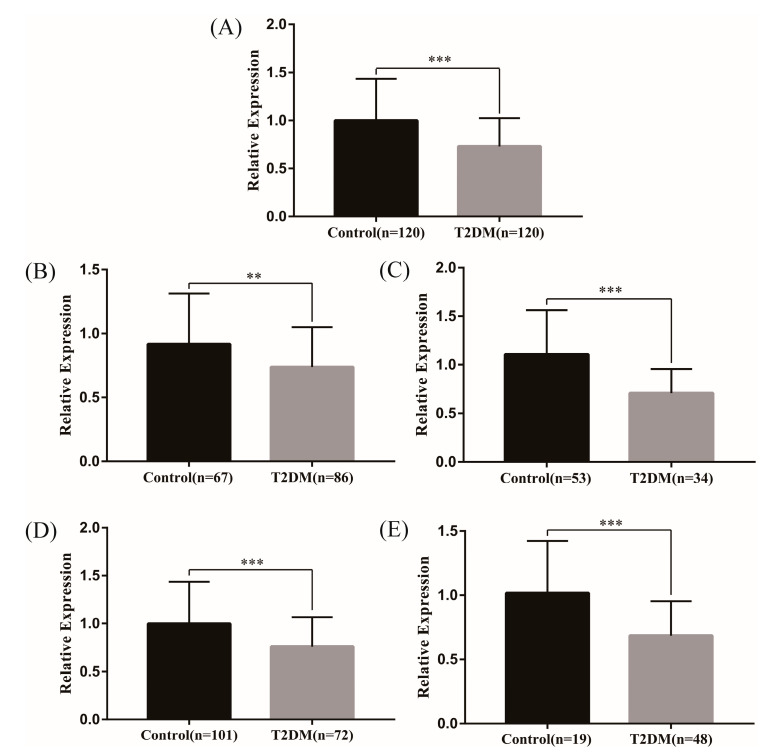
Expression of lncRNA ENST00000462455.1 between T2DM and control group ((**A**) in total (**B**) male (**C**) female (**D**) ≤60 (**E**) >60) (*** *p* < 0.001, ** *p* < 0.01).

Firstly, we analyzed the genes corresponding to the above 25 lncRNAs through the GSE 130,991 dataset, and a total of 13 genes were found in the dataset. Specially, the gene PLEKHM2, corresponding to the lncRNA ENST00000462455.1, was statistically significant (Table 2). Meanwhile, RT-qPCR was used to verify the expression levels of lncRNA ENST00000462455.1 in 120 hypertriglyceridemia T2DM patients and 120 hypertriglyceridemia patients with normal FPG. The results indicated that the expression level of ENST00000462455.1 in the T2DM subjects was decreased (*t* = 5.673, *p* < 0.001), and the same results were observed in gender and age subgroups (Figure 1). In addition, the ROC curve was used to assess the diagnostic power of ENST00000462455.1 (Figure S2 and Table S3).

### 2.2. Effects of lncRNA ENST00000462455.1 on the Activity and Function of MIN6 Cells

**Figure 2:**
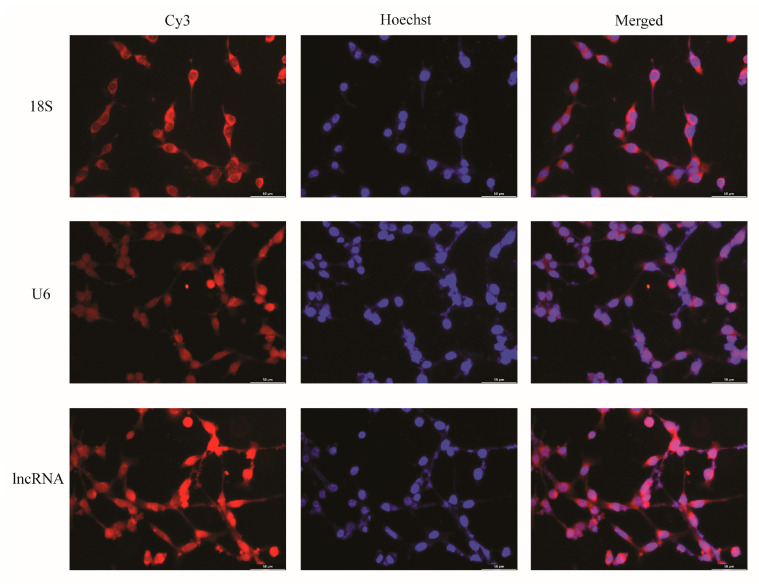
FISH localization of lncRNA ENST00000462455.1 in MIN6 cells (×40).

**Figure 3:**
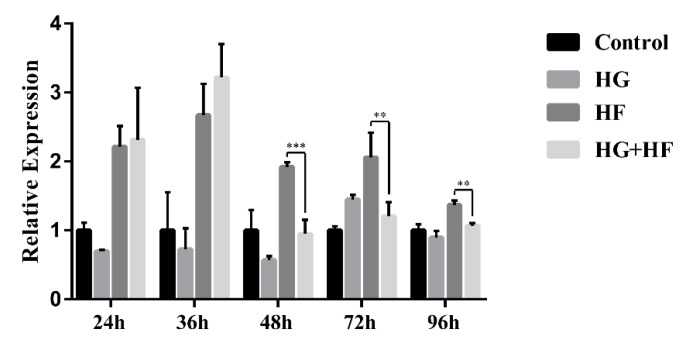
Expression of lncRNA ENST00000462455.1 in MIN6 cells under the circumstance of glycose and lipid (*** *p* < 0.001; ** *p* < 0.01)(*n* = 6 for each group).

Firstly, we detected the localization and distribution of ENST00000462455.1 in MIN6 cells by FISH. As internal reference genes, 18S was almost located in the cytoplasm and U6 was almost located in the nucleus. The results of the FISH indicated that the ENST00000462455.1 was distributed in both the cytoplasm and the nucleus (Figure 2). Next, we analyzed the expression of ENST 00000462455.1 in MIN6 cells cultured for 24 h, 36 h, 48 h, 72 h, and 96 h for the control, HG, HF, and HG + HF groups. The results indicated that, compared with the HF group, the expression level of ENST00000462455.1 in MIN6 cells in the HG + HF group decreased after 48 h (HF vs. HG + HF: 1.92 ± 0.05 vs. 0.95 ± 0.17, *p* < 0.001), 72 h (HF vs. HG + HF: 2.06 ± 0.29 vs. 1.21 ± 0.17, *p* < 0.01), and 96 h (HF vs. HG + HF: 1.37 ± 0.05 vs. 1.07 ± 0.03, *p* < 0.01) of culture in the corresponding environment (Figure 3).

**Figure 4:**
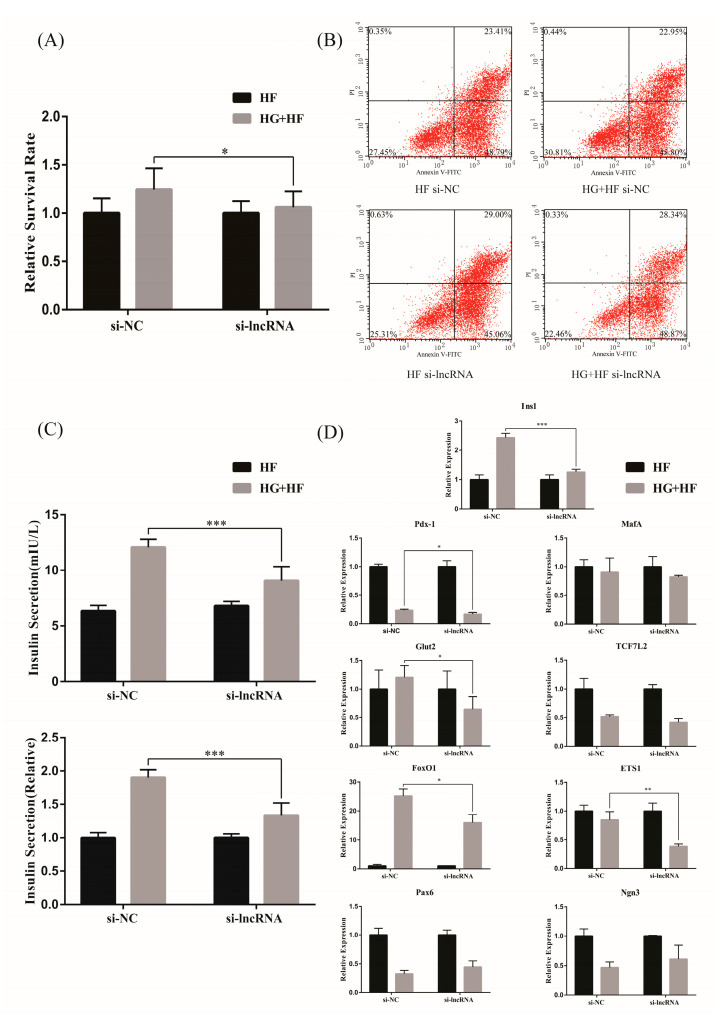
The effect of lncRNA ENST00000462455.1 on (**A**) the activity of MIN6 cells with CCK-8, (**B**) the apoptosis of MIN6 cells with flow cytometry, (**C**) the insulin secretion of MIN6 cells with ELISA, and (**D**) the transcription factors related to function and activity insulin secretion in MIN6 cells (*** *p* < 0.001, ** *p* < 0.01, * *p* < 0.05) (*n* ≥ 5 for each group).

To further explore the effect of lncRNA ENST00000462455.1 on the activity and function of MIN6, the siRNA against ENST00000462455.1 was transfected into MIN6 to silence the expression of the lncRNA. The results of RT-qPCR confirmed that the silencing effect was stable (Figure S3). Subsequently, we explored the effect of ENST00000462455.1 on MIN6 activity by the CCK-8 assay. Taking the HF group as a reference, we found that the relative survival rate of MIN6 in the HG + HF group with si-lncRNA was lower than that in the si-NC group (si-NC vs. si-lncRNA: 1.24 ± 0.21 vs. 1.06 ± 0.16, *p* < 0.05) (Figure 4A). Similarly, by flow cytometry, we observed that the relative apoptosis rate of MIN6 in the HG + HF group with si-lncRNA was higher than that in the si-NC group (Figure 4B). Meanwhile, the insulin level in the supernatant of the MIN6 cultured under the corresponding glycolipid environment for 48 h was detected by ELISA, thus assessing the effect of ENST00000462455.1 on the insulin secretion of MIN6. The results showed that the insulin secretion of MIN6 in the HG + HF group with si-lncRNA was lower than that in the si-NC group (si-NC vs. si-lncRNA: 12.06 ± 0.70 mIU/L vs. 9.07 ± 1.20 mIU/L, *p* < 0.001; si-NC vs. si-lncRNA(relative): 1.90 ± 0.11 vs. 1.33 ± 0.18, *p* < 0.001) (Figure 4C). In addition, RT-qPCR was also used to detect the expression levels of relevant key transcription factors. Taking the HF group as a reference, we found the expression levels of Ins1, Pdx-1, Glut2, FoxO1, and ETS1 in the HG + HF group with si-lncRNA were lower than those in the si-NC group (*p* < 0.05) (Figure 4D). Therefore, under a high-glucose and high-fat environment, the decreased expression of lncRNA ENST00000462455.1 could lead to the lowering of MIN6 cell activity and the occurrence of dysfunction.

### 2.3. Exploration of ceRNA Mechanism for lncRNA ENST00000462455.1

**Figure 5:**
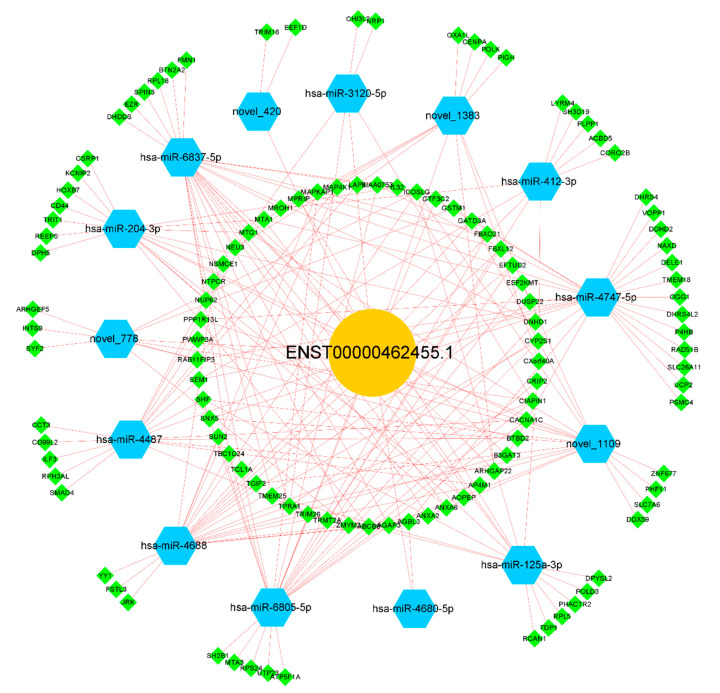
The ceRNA network of lncRNA ENST00000462455.1.

**Figure 6:**
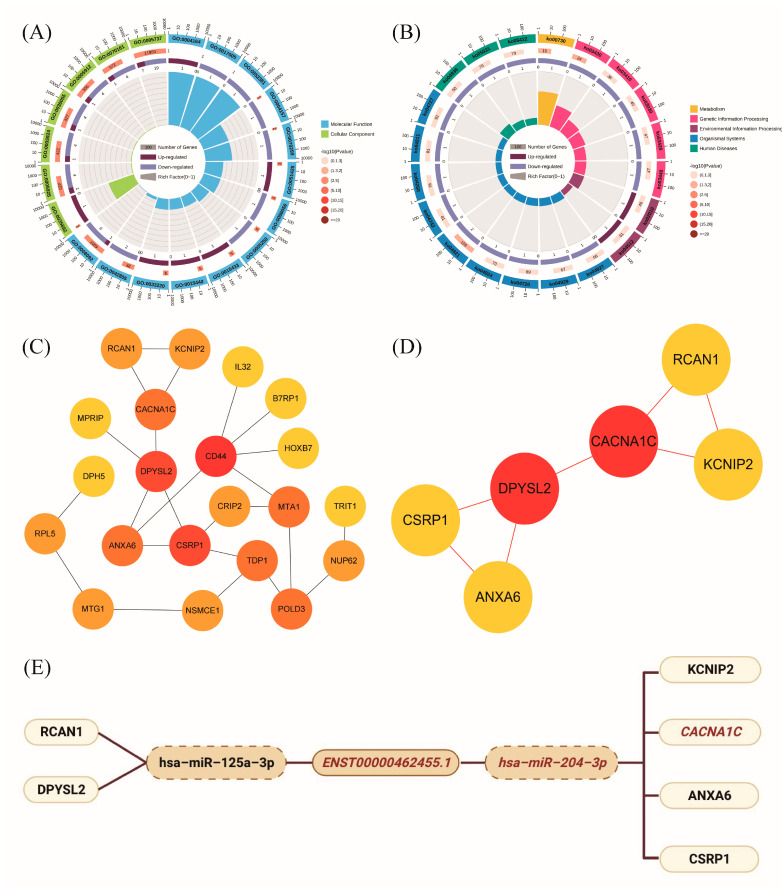
(**A**) GO enrichment analysis of key mRNAs in ceRNA networks; (**B**) KEGG enrichment analysis of key mRNAs in ceRNA networks; (**C**) PPI network of key mRNAs in ceRNA networks; (**D**) Key network module in PPI network; (**E**) The ceRNA regulatory axis of lncRNA ENST00000462455.1.

We further explored the possible mechanism of ENST00000462455.1 by constructing a ceRNA network which included lncRNA ENST00000462455.1 and its corresponding 14 miRNAs and 118 mRNAs (Figure 5). Given that miRNAs play an important role in the ceRNA network, we identified key miRNAs by searching the literature. Based on the available evidence, we found that miR-204-3p and miR-125a-3p were associated with type 2 diabetes or pancreatic β cells dysfunction, and 29 mRNAs corresponding to these two miRNAs were found in the ceRNA network (Table S4). Subsequently, GO and KEGG analysis were performed on these mRNAs (Figure 6A,B). The results indicated that CACNA1C, CSRP1, ANXA6, KCNIP2, and DPYSL2 are enriched in multiple pathways of BP, CC, and MF (Tables S5–S7). In particular, the results of the KEGG analysis showed that CACNA1C was enriched in multiple pathways including type 2 diabetes and insulin secretion (Table S8). Meanwhile, compared with the control group, the GSEA results found that CACNA1C was a core gene and the expression of it was decreased in hypertriglyceridemia subjects with T2DM (Table S9, Figure S4). In addition, we explored the interaction of key mRNAs in the ceRNA by establishing a PPI network, and a key network module was identified by cluster analysis: CSRP1-ANXA6-DPYSL2-CACNA1C-RCAN1-KCNIP2 (MCODE score: 2.8) (Figure 6C,D). Based on the above results, the possible ceRNA regulatory axis of lncRNA ENST00000462455.1 is shown in Figure 6E. Among them, ENST00000462455.1/miR-204-3p/CACNA1C may be the core regulatory axis.

## 3. Discussion

Protein-coding RNAs account for only about 2% of the genome [17,18]. Although noncoding RNAs do not have traditional RNA functions in protein translation, they have become novel basic regulators of gene expression. Existing evidence indicated that some lncRNAs in islet often map to the proximal end of related genes that related to function or development of pancreatic β cells and thus may have specific regulatory functions for the gene expression of pancreatic β cells [19,20,21]. In our study, transcriptome sequencing was first performed on whole blood samples of hypertriglyceridemia subjects with T2DM or normal FPG to get the differentially expressed lncRNAs. Subsequently, the differentially expressed lncRNA ENST00000462455.1 was verified by GEO and RT-qPCR, and its potential value in clinical settings was also assessed via ROC. In addition, compared with the HF environment, we found that the expression of ENST00000462455.1 in MIN6 cells decreased under the HG + HF environment. Therefore, lncRNA ENST00000462455.1 was viewed as a differentially expressed lncRNA in hypertriglyceridemia patients with T2DM and normal FPG.

We further explored the effect of ENST00000462455.1 on the function and activity of MIN6 cells. After silencing ENST00000462455.1, we found that the activity of MIN6 cells decreased and the apoptosis rate increased. Meanwhile, the insulin secretion was also reduced. In addition, the expression levels of transcription factors, including Ins1, Pdx-1, Glut2, FoxO1, and ETS1, were decreased after silencing ENST00000462455.1. As an inherent regulatory gene of insulin, Ins1 is regulated by circulating levels of glucose and plays an important role in maintaining mature pancreatic β cells mass and function, insulin secretion and reserve, and glucose homeostasis [22,23]. Similarly, the function of Pdx-1 is to maintain mature islet function, mass, and the regeneration of pancreatic β cells [24]. Meanwhile, Pdx-1 may also be a key factor related to the adverse effects of lipid metabolism disorders on pancreatic islets [25]. FoxO1 could regulate the proliferation, apoptosis, and differentiation of pancreatic β cells and play a role in insulin secretion and resistance to oxidative stress [26]. Simultaneously, FoxO1 is closely related to Ins1 and Pdx-1. Previous study found that FoxO1 transgenic mice significantly elevated the expression levels of Ins1 and Pdx-1 [27]. In fact, the relationship between FoxO1 and Pdx-1 has been confirmed during the development of the body. FoxO1 can activate itself in the early stage of pancreatic development by mediating the expression of Pdx-1 [28]. Specially, although the function of Glut2 is merely to catalyze the passive transport of glucose across plasma membranes, this transport activity is important for the control of cellular mechanisms impinging on gene expression, the regulation of intracellular metabolic pathways, and the induction of hormonal and neuronal signals, which together form the basis of an integrated interorgan communication system to control glucose homeostasis [29]. In addition, previous study also found that the overexpression of Ets-1 in MIN6 cells could protect them from severe hypoxic injury in a mitochondria-dependent method [30].

One of the main mechanisms of lncRNAs is that they can become endogenous competing RNAs for miRNAs affecting the expression of downstream genes, thereby promoting or inhibiting the development of diseases. In our study, ENST00000462455.1 was observed in both the cytoplasm and nucleus by FISH. Existing evidence indicated that lncRNAs stably expressed in the cytoplasm are ideal ceRNAs (although recent studies also found that some nuclear-localized lncRNAs could also act as ceRNAs). Therefore, we further constructed the ceRNA network of ENST00000462455.1 by the bioinformatics method and found that ENST00000462455.1/miR-125a-3p/RCAN1/DPYSL2 may be one of the regulatory axes. Previous studies have shown that miR-125a-3p could inhibit the expression of insulin receptors via the insulin signaling pathway, resulting in insulin resistance, thus leading to lipid and carbohydrate metabolism disorder [31]. Meanwhile, miR-125a-3p is also related to diabetic cardiomyopathy and diabetic nephropathy [32]. RCAN1 has a role in the pancreatic β cell dysfunction for T2DM [33]. Some studies found that the acute induction of RCAN1 by increased reactive oxygen species and hyperglycemia could inhibit endocrine cell apoptosis and protect them from damage. However, some evidence indicated that chronic overexpression of RCAN1 could also adversely affect cells, leading to pathological changes in neurons and endocrine cells associated with T2DM [33]. Therefore, more studies for the molecular mechanisms of RCAN1 need to be performed.

Another possible ceRNA regulatory axis is ENST00000462455.1/miR-204-3p/KCNIP2/CACNA1C/ANXA6/CSRP1. Among them, ENST00000462455.1/miR-204-3p/CACNA1C may be the core regulatory axis. Previous studies found that the expression of miR-204 is increased in pancreatic islets of T2DM and elevated serum miR-204 is a marker of ongoing pancreatic β cell death [34]. Meanwhile, miR-204 can directly target and inhibit the endoplasmic reticulum transmembrane factor protein kinase R-like endoplasmic reticulum kinase (PERK) and its downstream signaling pathways, thereby aggravating ER-stress-induced pancreatic β cell apoptosis [35]. As a chain of miR-204, miR-204-3p is involved in various diabetic complications. In diabetic cataract, miR-204-3p can regulate the migration and epithelial-to-mesenchymal transition in lens epithelial cells [36]. Meanwhile, miR-204-3p also plays a role in high-glucose-induced podocyte apoptosis and dysfunction [37]. In addition, for diabetic cardiomyopathy, miR-204-3p can regulate cardiomyocyte autophagy, thus affecting myocardial ischemia/reperfusion injury [38].

Voltage-gated calcium channels (VGCCs) and potassium channels are important to insulin secretion [39,40,41]. Among them, the L-type voltage-gated calcium channels (LVGCCs) are present in pancreatic β cells and are involved in glucose transport, lipolysis, and lipogenesis [42,43]. Although LVGCCs account for only ∼50% of the total Ca^2+^ current, their inhibition reduces glucose-induced insulin secretion by 80% and nearly abolishes insulin release in vivo [44]. In humans, the two main LVGCCs are Ca_v_1.2 and Ca_v_1.3, and CACNA1C is the encoding gene of Ca_v_1.2. It was found that Ca_v_1.2 was required for first-phase insulin secretion and rapid exocytosis in pancreatic β cells, and the expression level of CACNA1C was also high in the cells [45,46]. In mice, Ca_v_1.2 was the only LVGCC and the knockout of CACNA1C was lethal (glucose intolerance and loss of first-phase insulin secretion were observed) [47]. In addition, CACNA1C is also involved in diabetic peripheral neuropathy, diabetic heart disease, and diabetic cataract [48,49,50]. KCNIP2 (encodes the KChIP2 protein) interacts with the subfamily of the voltage-gated potassium channel to increase the current density, accelerate the recovery from inactivation, and slow inactivation kinetics [51]. Existing evidence indicated that the lack of insulin signaling in the heart of T2DM patients may be one of the mechanisms for the decreased expression of KCNIP2, which in turn leads to abnormal changes in cardiac electrophysiology [52]. In addition, ANXA6 is involved in cholesterol transport, accumulation, and storage of TG, and plays an important role in the glucose and lipid balance by regulating the release of adiponectin [53,54,55].

Some limitations exist in this study. We used MIN6 cells, a mouse pancreatic beta cell line, for the experimental verification of lncRNA ENST00000462455.1 functions. Considering the species difference, the effect of this lncRNA on T2DM of human needs further evaluation. Moreover, the study lacked corresponding animal model verification. Meanwhile, our study only used bioinformatics methods to explore the possible ceRNA regulatory mechanism of ENST00000462455.1, and further experimental verification is required.

## 4. Materials and Methods

### 4.1. Participants

Six newly diagnosed T2DM patients and six patients with normal blood glucose were recruited to perform RNA sequencing. All subjects were Han Chinese, aged 40–65 years, and were recruited at the First Hospital of Jilin University from July to September 2020. Patients were diagnosed based on the guidelines for the prevention and control of type 2 diabetes in China (2017 Edition): Patients with type 2 diabetes were defined as fasting plasma glucose (FPG) ≥ 7.0 mmol/L or oral glucose tolerance test (OGTT) two-hour blood glucose ≥ 11.1 mmol/L. FPG < 6.1 mmol/L and OGTT < 7.8 mmol/L were defined as the normal controls. Meanwhile, the level of triglycerides (TG) in all participants was ≥1.7 mmol/L according to the guidelines for prevention and treatment of dyslipidemia in China (2016 Edition). All patients had not previously controlled their blood glucose through drugs or other treatments. Moreover, the corresponding genes of the lncRNAs were verified via the GSE 130,991 dataset (910 samples). A total of 92 T2DM and 96 controls with hypertriglyceridemia were selected from the dataset based on the above guidelines. Meanwhile, we also collected 120 T2DM and 120 controls with hypertriglyceridemia to perform RT-qPCR validation at the First Hospital of Jilin University from July to August 2021.All patients with a history of coronary artery disease (CAD), hypertension, atrial fibrillation, myocardial infarction, tumor, acute infectious disease, immune disease, and hematological disease were excluded from the study. All participants provided written informed consent and the study was approved by Ethics Committee of the Public Health of the Jilin University, and the privacy of the participants are strictly confidential.

### 4.2. RNA Sequencing

Total RNA in blood was isolated and purified using a total RNA extraction kit. The NanoPhotometer^®^ spectrophotometer (IMPLEN, Westlake Village, CA, USA) and RNA Nano 6000 Assay Kit of the Agilent Bioanalyzer 2100 system (Agilent Technologies, Santa Clara, CA, USA) were used to assess the RNA purity and integrity, respectively. The chain-specific library was constructed by removing the ribosomal RNA. After the library was qualified, Illumina PE150 sequencing was performed according to the pooling of the effective concentration of the library and the data output requirements. Followed by the sequencing, data filtering was conducted: we removed reads with adapter and N (N means that the nucleobase information cannot be determined) ≥ 0.002, and the paired reads that contain low-quality nucleobases (>50%) in single-end reads were also removed. Meanwhile, the Q20, Q30, and GC content were calculated, and the clean reads were obtained. Subsequently, the mapping analysis was performed by the software Hisat2 for the corresponding clean reads. The reference database was GRCh38.p12 (human) and GRCm38.p6 (mouse). Based on the mapping results, we further assembled, filtered, and quantified the transcripts by using the Stringtie and Cuffmerge software. Finally, the expression level matrix was obtained. All analyses in the study were based on the data and the data could be found in GEO database (GSE193436).

### 4.3. Real-Time Quantitative Polymerase Chain Reaction (RT-qPCR)

The total RNA was extracted using the MolPure^®^ Blood RNA Kit (19241ES50, YEASEN) or MolPure^®^ Cell RNA Kit (19231ES50, YEASEN) based on the sample type. Subsequently, we used the lnRcute lncRNA First-Strand cDNA Kit (KR202, TIANGEN) or FastKing gDNA Dispelling RT SuperMix (KR118, TIANGEN) to conduct reverse transcription. The cDNA was then analyzed by RT-qPCR using lnRcute lncRNA qPCR Kit (FP402, TIANGEN) or SuperReal PreMix Plus (SYBR Green) (FP205, TIANGEN) on the QuantStudio 3 system (Applied Biosystems, Waltham, MA, USA). The PCR primers are shown in Table S10. Expression data were normalized to the expression of β-actin with the 2^−ΔΔCt^ method.

### 4.4. Cell Culture

MIN6 cells (mouse pancreatic beta cell line) were cultured in RPMI Medium 1640 (31800, Solarbio, Beijing, China) supplemented with 10% fetal bovine serum (FBS) (04-001-1A, Biological Industries, Cromwell, CT, USA) at 37 °C with 5% CO_2_.

### 4.5. Fluorescence In Situ Hybridization (FISH)

RiboTM lncRNA FISH Probe Mix (lnc11001001, RIBOBIO) and RiboTM Fluorescent in Situ Hybridization Kit (C10910, RIBOBIO) were used for the FISH of lncRNA, thus detecting the distribution of the target lncRNA. The cell slides were placed at the bottom of a 24-well plate and each well was plated with 1 × 10^5^ cells. After the cells had grown to about 80%, the cells were washed with phosphate-buffered saline (PBS) and fixed with 4% paraformaldehyde. Subsequently, the cells were washed again and treated with permeabilization solution, then 200 μL of prehybridization solution was added and the cells were blocked at 37 °C for 30 min. The prehybridization solution was discarded and 100 μL of the hybridization solution containing the lncRNA FISH probe was added for overnight hybridization at 37 °C. Next day, the cells were washed by PBS and stained with DAPI and photographed by fluorescence microscopy, with 18S and U6 as the reference genes.

### 4.6. Construction of Cellular Environment and Determination of lncRNA Expression

Based on different glycolipid environments, our experiment was divided into four experimental groups: control (5 mmol/L D-glucose + PBS), high glucose (HG) (30 mmol/L D-glucose + PBS), high fat (HF) (5 mmol/L D-glucose + 400µmol/L sodium palmitate), and high glucose and high fat (HG + HF) (30 mmol/L D-glucose + 400µmol/L sodium palmitate) [56,57]. The expression of the target lncRNA in each group was determined by qRT-PCR after 24 h, 36 h, 48 h, 72 h, and 96 h.

### 4.7. Cell Transfection

The siRNA was transfected by liposome reagent transfection to silence the target lncRNA. Corresponding sequence of siRNA was shown in Table S11. Firstly, six-well plates were seeded with 2 × 10^5^ cells per well. After 24 h, siRNA against target lncRNA (GenePharma) was transfected into cells by using Lipofectamine 2000 (11668019, Invitrogen). After incubation at 37 °C with 5% CO_2_ for 6 h, the medium was changed to complete medium (supplemented with 10% FBS) for another 24 h. Subsequently, the RNA in the cells was directly extracted or further cultivated in different glycolipid environments for 48 h and the expression level of the target lncRNA in the negative control group (si-NC) and experimental group (si-lncRNA) was detected to evaluate the effect of transfection.

### 4.8. CCK-8 Assay

The cells were seeded in 96-well plates (4 × 10^3^ cells per well). After the lncRNA was silenced, corresponding glycolipid environment were constructed for 48 h, and then 10μL of CCK-8 reagent (CK04, Dojindo) was added to each well. Subsequently, the plate was incubated for another 1–4 h and the absorbance values were measured at 450 nm with an enzyme-linked immunometric meter.

### 4.9. Apoptosis Assay

Cell apoptosis was detected by the FITC Annexin V Apoptosis Detection Kit I (556547, BD BIOSCIENCES PHARMINGEN). Firstly, cells were seeded in 6-well plates (2 × 10^5^ cells per well). After the lncRNA was silenced, the corresponding glycolipid environments were constructed for 48 h. Then, the original medium in the plate was discarded and cold PBS was added to wash the cells. Subsequently, 1 × binding buffer was added to each well and the cells were stained with FITC and PI. After 15 min incubation protecting from light, flow cytometry analysis was performed by using a FACSCalibur (BD BIOSCIENCES PHARMINGEN).

### 4.10. Enzyme-Linked Immunosorbent Assay (ELISA)

Insulin secretion was assessed by ELISA. Similarly, cells were seeded in 6-well plates (2 × 10^5^ cells per well). After the lncRNA was silenced, the corresponding glycolipid environments were constructed for 48 h. Then, the supernatant was collected and detected by Mouse INS ELISA kit (ml001983, mlbio). All experiments were performed strictly in accordance with the manufacturer’s instructions.

### 4.11. Detection of Transcription Factor Levels of Pancreatic β Cell Function and Activity

Cells were seeded in 6-well plates (2 × 10^5^ cells per well). After the lncRNA was silenced, corresponding glycolipid environments were constructed for 48 h. Subsequently, RT-qPCR was used to detect the transcription factors of pancreatic β cell function and activity (Ins1, Pdx-1, MafA, Glut2, TCF7L2, FoxO1, ETS1, Pax6, Ngn3).

### 4.12. Statistical Analysis

Normal continues variables were described by mean and standard deviation. Meanwhile, median and interquartile ranges were used to describe the skewed continues variables. Correspondingly, the t-test and Wilcoxon rank-sum test were conducted based on the data distribution. Chi-square test was conducted for categorical variables. One-way ANOVA was used for comparison among multiple groups, and LSD was performed for pairwise comparison. The diagnostic value of the lncRNA for T2DM in hypertriglyceridemia subjects was evaluated by the ROC curve. All above analyses were mainly performed by SPSS 24.0 and GraphPad Prism 7.0 software. A 2-sided *p* value less than 0.05 was considered significant. Independent replicated experiments were conducted in our study.

R 4.0.4, Cytoscape 3.8.2 and GSEA 4.2.1 software were used to conduct bioinformatics analysis. Differentially expressed genes were screened using the limma package [58] and the correlation between genes was analyzed by Pearson correlation. Meanwhile, the ggplot2 [59] and pheatmap [60] packages were used to draw the volcano plot and heat map, respectively. The ceRNA network construction strategy of the target lncRNA is shown in Figure S5, and Cytoscape was used to draw the networks. The clusterProfiler package [61] was used for GO (including Biological Process (BP), Cellular Component (CC), and Molecular Function (MF)) and KEGG enrichment analysis, and corresponding enrichment circle maps were drawn via the online analysis tool (https://www.omicsshare.com/tools/, accessed on 13 November 2021). Gene Set Enrichment Analysis (GSEA) was performed using GSEA software. In addition, PPI network analysis was performed by STRING 11.5 (http://string-db.org, accessed on 12 November 2021) and Cytoscape, and the MCODE was used to conduct cluster analysis in PPI network.

## 5. Conclusions

The lncRNA ENST00000462455.1 is a potential biomarker for hypertriglyceridemia patients with T2DM. More experimental studies are needed to verify the function of the lncRNA and analyze its possible mechanism.

## Figures and Tables

**Table 1:** Differentially expressed lncRNA between the two groups (*p*-adj ≤ 0.05).

Transcript Id	log_2_FC	*p*	*p*-adj	Regulation
TCONS_00281181	13.77101282	2.68 × 10^−5^	0.026712	up
ENST00000420364.1	13.56976713	3.23 × 10^−5^	0.029493	up
ENST00000358888.7	13.05634787	1.62 × 10^−5^	0.02084	up
TCONS_00333304	12.93059465	6.24 × 10^−8^	0.000966	up
ENST00000437561.2	12.91469606	3.00 × 10^−6^	0.008758	up
ENST00000515602.5	12.59459795	8.01 × 10^−8^	0.001117	up
ENST00000485760.5	12.30560805	1.03 × 10^−5^	0.018221	up
ENST00000595118.5	11.98997501	4.24 × 10^−7^	0.00296	up
ENST00000663944.1	11.31194966	4.01 × 10^−5^	0.033094	up
ENST00000558173.5	7.728315847	6.05 × 10^−5^	0.042577	up
ENST00000444301.5	−13.7301692	2.75 × 10^−5^	0.027059	down
ENST00000472023.5	−13.0473268	4.68 × 10^−5^	0.03701	down
TCONS_00414967	−12.8104008	7.27 × 10^−5^	0.046655	down
ENST00000668922.1	−12.754318	5.95 × 10^−6^	0.013425	down
TCONS_00242343	−12.7213404	5.81 × 10^−6^	0.013296	down
TCONS_00333305	−12.7081243	4.37 × 10^−6^	0.010915	down
ENST00000621798.4	−11.9489468	1.62 × 10^−5^	0.02084	down
ENST00000664414.1	−11.4516783	1.44 × 10^−6^	0.006079	down
ENST00000600527.5	−11.411395	3.53 × 10^−5^	0.030778	down
ENST00000462455.1	−11.3309579	3.36 × 10^−5^	0.030145	down
ENST00000521800.2	−10.9959184	5.41 × 10^−5^	0.040101	down
TCONS_00163822	−10.3010495	1.86 × 10^−5^	0.022399	down
TCONS_00320824	−9.76143147	2.77 × 10^−5^	0.027059	down
ENST00000424094.6	−9.36711252	4.91 × 10^−5^	0.038003	down
ENST00000482484.1	−9.19090354	5.56 × 10^−5^	0.040218	down

**Table 2:** Validation of gene expression via GSE130991.

lncRNA ID	lncRNA Gene Symbol	*p*
ENST00000595118.5	LTBP4	0.736
ENST00000485760.5	DAB1	0.001
ENST00000358888.7	RPL41	0.906
TCONS_00163822	CA10	0.985
TCONS_00281181	SUMF1	0.002
ENST00000420364.1	LINC00189	0.849
ENST00000462455.1	PLEKHM2	0.024
ENST00000472023.5	BACH2	0.420
ENST00000424094.6	GNAS-AS1	0.223
ENST00000521800.2	ARSB	0.368
ENST00000482484.1	CDC73	0.379
ENST00000558173.5	TRIM69	0.708
TCONS_00414967	NCBP2L	0.298

## Data Availability

The clean sequencing data have been uploaded into the GEO database (GSE193436) and other data for the study are available from the corresponding author on a reasonable request.

## Supplementary Materials

The following supporting information can be downloaded at: https://www.mdpi.com/article/10.3390/ijms24054279/s1.
