# Supplementary material for: The Association between the Differential Expression of lncRNA and Type 2 Diabetes Mellitus in People with Hypertriglyceridemia
Source: Int J Mol Sci. 2023 Feb 21;24(5):4279. doi: 10.3390/ijms24054279 (PMC10002095; doi:10.3390/ijms24054279)
Supplement: Supplementary file 1 [file ijms-24-04279-s001.zip › Table S1.pdf]

Table S1 Basic situation of T2DM group and control group (n=12)

Characteristic	T2DM (n=6)	Control (n=6)	$\chi^2/t/z$	*P*
Age(year)	54.00±6.03	52.00±6.26	-0.563	0.586
Weight(kg)	65.00(61.25, 75.00)	60.00(58.75, 77.50)	-0.491	0.624
BMI(kg/m^2)	22.62±2.49	24.10±2.70	0.981	0.350
TC(mmol/L)	4.86±0.92	5.94±1.20	1.755	0.110
LDL(mmol/L)	2.65±0.52	3.52±0.56	2.811	0.018
HDL(mmol/L)	1.09(0.85, 1.23)	1.31(1.10, 2.11)	-1.684	0.092
TG(mmol/L)	2.59(2.24, 6.51)	2.29(1.98, 3.24)	-0.961	0.337
Creatinine (μmol/L)	63.20±7.26	68.00±13.29	0.709	0.498
Uric acid (μmol/L)	352.50±40.00	399.00±116.84	0.752	0.476
ALT(U/L)	18.75±0.96	19.00±4.08	0.119	0.909
AST(U/L)	22.00(19.00, 24.25)	23.00(16.25, 24.50)	-0.441	0.659
FPG (mmo/L)	8.80(7.95, 13.15)	5.25(4.88, 5.50)	-2.892	0.004

BMI: Body Mass Index; TC: total cholesterol; LDL: low-density lipoprotein; HDL: High-density lipoprotein; TG: triglyceride; ALT: alanine aminotransferase; AST: aspartate aminotransferase; FPG: fasting plasma glucose
