# Supplementary material for: The Association between the Differential Expression of lncRNA and Type 2 Diabetes Mellitus in People with Hypertriglyceridemia
Source: Int J Mol Sci. 2023 Feb 21;24(5):4279. doi: 10.3390/ijms24054279 (PMC10002095; doi:10.3390/ijms24054279)
Supplement: Supplementary file 1 [file ijms-24-04279-s001.zip › Table S10.pdf]

Table S10 primer sequences of lncRNA and transcription factors

lncRNA/ transcription factors	primer sequences
TCONS_00333304	F: 5’-AGAGACCTGGCTGCTGTGT-3’
	R: 5’-CCAATGCAAAGGCACTAATGGG-3’
ENST00000515602.5	F: 5’-AAGCCACCAAACATCCCAGTTACC-3’
	R: 5’-TCCTGCTGCCTCCTCTGAAGAC-3’
ENST00000595118.5	F: 5’-CGGAATGTGACATGGCAGGAGTG-3’
	R: 5’-CGTGAGGGCACAATGACTGGTAC-3’
ENST00000664414.1	F: 5’-ACAACTCAAATCCCAGGAACACAGG-3’
	R: 5’-CAGCATCCACCTCCACTCCAATTC-3’
ENST00000437561.2	F: 5’-ACACCACCAGAAGCCGAGAGAG-3’
	R: 5’-GTCAGGACGCACGCTGGATTAG-3’
TCONS_00333305	F: 5’-TTGCGCTCCGTAGTGGACTC-3’
	R: 5’-CCTCTCAATGCAACGGCTTCA-3’
TCONS_00242343	F: 5’-GTGGTGAAGTTTGGGCCTTTTGTG-3’
	R: 5’-CTCCTGGGACTGGTTTTCTCTGTTG-3’
ENST00000668922.1	F: 5’-GGTCCGCCCGTTTCTCTAATCAAG-3’
	R: 5’-TTCTCAATGGAAGCGTTCGTGTCTC-3’
ENST00000485760.5	F: 5’-GTCAGGATCGCAGTGAAGCC-3’
	R: 5’-CCGAGCTGCGGAAACTTCAT-3’
ENST00000358888.7	F: 5’-TCCGCCATACCTCCTGAACTACTG-3’
	R: 5’-GCATTCGCTTCTTCCTCCACTTCTC-3’
ENST00000621798.4	F: 5’-CATCACCAATCCGTCGCCAGAAG-3’
	R: 5’-AGGAGGAGGAGGAGGAGGAAGAG-3’
TCONS_00163822	F: 5’-GCACTGAGACGAAGAGCTGATGAAG-3’
	R: 5’-TGGGATTCTGGAGAGCACTGAAGG-3’
TCONS_00281181	F: 5’-AATAGGAGTGGTGAGAGAGGGCATC-3’
	R: 5’-GACAAACCCACAGCCAATAGCATAC-3’
ENST00000444301.5	F: 5’-GCCTCTCCCTGACAAGCAAAGTG-3’
	R: 5’-AGTCGGCAGAGCCAGTCTGATAG-3’
TCONS_00320824	F: 5’-CTCCTCTCCTTTCCCTCCCAGAAC-3’
	R: 5’-TTCCACCACTCCACATGCAAGAAC-3’
ENST00000420364.1	F: 5’-ACTCCTGTGCTGAAACTTGCCTTAG-3’
	R: 5’-CCTGTTGTTCCCAGTGGTTACTCC-3’
ENST00000462455.1	F: 5’-CAGTACCTGCTGGACTTTGAAGACC-3’
	R: 5’-CGGAGTTGAAAGAGTTGAGGGACAG-3’
ENST00000600527.5	F: 5’-ACCTGATGCTATGTGCGCTG-3’
	R: 5’-AAGGGCTTCTTCTGTTGCCC-3’
ENST00000663944.1	F: 5’-AGACAGTAGGAGGTTGGCAGATGG-3’
	R: 5’-ACAGTCCTCTCCAATGGGTGATCC-3’
ENST00000472023.5	F: 5’-CAGAAAGTGAGGGCTCCAGGAATG-3’
	R: 5’-GGTCAGTGAGTGTCCACCTTGTTC-3’

ENST00000424094.6	F: 5’-GGAGAGGGCTTACTGCGAGGAG-3’
	R: 5’-GTGGTTGGATGCTTTTGTGGTCTTC-3’
ENST00000521800.2	F: 5’-GGGTGGAATGACCTAGTGCAATGG-3’
	R: 5’-ACCTGGAGTGGGATGAAGAGATGG-3’
ENST00000482484.1	F: 5’-TTGAGAAGCCCAAAGGAAAGGACTG-3’
	R: 5’-TGCACGTCGGACATAAACAGGATG-3’
ENST00000558173.5	F: 5’-GGGAAAGCCTTGAATGAGGAGATGG-3’
	R: 5’-TGCCTGAATGCTCACCAACATATCC-3’
TCONS_00414967	F: 5’-CTACCCTTCTCCTTTGCCAGTTGC-3’
	R: 5’-CCAAGCCAGTGAGCCTCTAATGC-3’
ENST00000462455.1(mouse)	F: 5’-TGGAGAGCTACCTGCGTCTGTTC-3’
	R: 5’-ACACCAAGGTCAGGAAGAGAGTCAG-3’
Ins1	F: 5’-GTGGAACAACTGGAGCTGGG-3’
	R: 5’-CTGATCCACAATGCCACGCT-3’
Pdx-1	F: 5’-GTTCCAAAACCGTCGCATGAAGTG-3’
	R: 5’-CCGAGGTCACCGCACAATCTTG-3’
MafA	F: 5’-CGGAGGAACAGAAGGAGGAGGAG-3’
	R: 5’-CTGCTGTCAACTCTGGGCTGTG-3’
Glut2	F: 5’-CCTTCAGCAACTGGGTCTGC-3’
	R: 5’-CAGGGTGAAGACCAGGACCA-3’
TCF7L2	F: 5’-CCTCTGCCCTCTGTCCCAATGG-3’
	R: 5’-TTGATGGTTGTGCGAGCGATGAG-3’
FoxO1	F: 5’-ACATCTGCCATGAACCGCTTGAC-3’
	R: 5’-CACCCATCCTACCATAGCCATTGC-3’
ETS1	F: 5’-TCGGCATCATAGCACAGTTCAAGTC-3’
	R: 5’-CTGAGTCTTCCATGTTTCGGGTAGC-3’
Pax6	F: 5’-GAGTGTCAGTTCCCGTCCAAGTTC-3’
	R: 5’-TCCTCTCTCGATCACATGCTCTCTC-3’
Ngn3	F: 5’-TAGCAGAACTTCAGAGGGAGCAGAG-3’
	R: 5’-CAGTGACAAGCAGCAGTGGATAGG-3’
