# Supplementary material for: The Association between the Differential Expression of lncRNA and Type 2 Diabetes Mellitus in People with Hypertriglyceridemia
Source: Int J Mol Sci. 2023 Feb 21;24(5):4279. doi: 10.3390/ijms24054279 (PMC10002095; doi:10.3390/ijms24054279)
Supplement: Supplementary file 1 [file ijms-24-04279-s001.zip › Table S11.pdf]

Table S11 Sequences of si-lncRNA and its negative control

	primer sequences
si-lncRNA	F: 5’- GGUUGGAGUUUAUUCGGUUTT -3’
	R: 5’- AACCGAAUAAACUCCAACCTT -3’
si-NC	F: 5’- UUCUCCGAACGUGUCACGUTT -3’
	R: 5’- ACGUGACACGUUCGGAGAATT -3’
