# Supplementary material for: The Association between the Differential Expression of lncRNA and Type 2 Diabetes Mellitus in People with Hypertriglyceridemia
Source: Int J Mol Sci. 2023 Feb 21;24(5):4279. doi: 10.3390/ijms24054279 (PMC10002095; doi:10.3390/ijms24054279)
Supplement: Supplementary file 1 [file ijms-24-04279-s001.zip › Table S2.pdf]

Table S2 Summary of data from RNA sequencing.

Group	Sample	Raw reads	Clean reads	Raw bases(G)	Clean bases(G)	Error rate(%)	Q20(%)	Q30(%)	GC content(%)
T2DM	HTG_D_1	95930240	93380952	14.39	14.01	0.02	98.25	95.03	58.36
	HTG_D_2	95047612	93684148	14.26	14.05	0.02	98.08	94.78	60.28
	HTG_D_3	87308266	85200132	13.10	12.78	0.02	98.14	94.77	56.51
	HTG_D_4	93872544	92111104	14.08	13.82	0.03	97.60	93.34	59.32
	HTG_D_5	98171594	95680364	14.73	14.35	0.02	98.03	94.36	59.55
	HTG_D_6	92939324	90560052	13.94	13.58	0.03	97.77	93.84	56.14
Control	HTG_N_1	85958252	84363446	12.89	12.65	0.02	98.32	95.41	60.36
	HTG_N_2	85005964	83070364	12.75	12.46	0.02	97.83	94.44	61.92
	HTG_N_3	91876150	89511812	13.78	13.43	0.02	97.92	94.57	59.15
	HTG_N_4	91519536	89611444	13.73	13.44	0.02	98.14	94.97	57.04
	HTG_N_5	91153458	89365918	13.67	13.40	0.02	98.20	95.04	58.43
	HTG_N_6	84993186	83520980	12.75	12.53	0.02	98.21	95.01	57.19
