# Supplementary material for: The Association between the Differential Expression of lncRNA and Type 2 Diabetes Mellitus in People with Hypertriglyceridemia
Source: Int J Mol Sci. 2023 Feb 21;24(5):4279. doi: 10.3390/ijms24054279 (PMC10002095; doi:10.3390/ijms24054279)
Supplement: Supplementary file 1 [file ijms-24-04279-s001.zip › Table S3.pdf]

Table S3 ROC curve of lncRNA ENST00000462455.1 expression

	Sensitivity (%)	Specificity (%)	AUC	*P*	95%CI
Total	66.7	62.5	0.696	＜0.001	0.631-0.762
Male	37.2	83.6	0.636	0.004	0.549-0.724
Female	58.8	86.8	0.774	＜0.001	0.676-0.872
≤60	61.1	65.3	0.667	＜0.001	0.586-0.747
＞60	45.8	94.7	0.769	0.001	0.650-0.888
