# Supplementary material for: The Association between the Differential Expression of lncRNA and Type 2 Diabetes Mellitus in People with Hypertriglyceridemia
Source: Int J Mol Sci. 2023 Feb 21;24(5):4279. doi: 10.3390/ijms24054279 (PMC10002095; doi:10.3390/ijms24054279)
Supplement: Supplementary file 1 [file ijms-24-04279-s001.zip › Table S4.pdf]

Table S4 Key miRNA and its associated mRNA in the ceRNA network

miRNA	mRNA	miRNA	mRNA
hsa-miR-125a-3p	MPRIP	hsa-miR-204-3p	IL32
	NTPCR		AOPEP
	SHF		MTG1
	TMEM25		ABCB9
	ABCB9		CACNA1C
	ICOSLG		NSMCE1
	CRIP2		DNHD1
	MTA1		MTA1
	TDP1		ANXA6
	POLD3		NUP62
	DPYSL2		CD44
	PHACTR2		TRIT1
	RPL5		DPH5
	RCAN1		KCNIP2
			REEP5
			CSRP1
			HOXB7
