# Supplementary material for: The Association between the Differential Expression of lncRNA and Type 2 Diabetes Mellitus in People with Hypertriglyceridemia
Source: Int J Mol Sci. 2023 Feb 21;24(5):4279. doi: 10.3390/ijms24054279 (PMC10002095; doi:10.3390/ijms24054279)
Supplement: Supplementary file 1 [file ijms-24-04279-s001.zip › Table S5.pdf]

Table S5 GO enrichment analysis of mRNAs in ceRNA networks (top 20 pathways of BP)

GO ID	Term	Input
GO:0044057	regulation of system process	KCNIP2;MTG1; TMEM25; CACNA1C; ANXA6
GO:0006301	postreplication repair	POLD3; NSMCE1
GO:0034346	positive regulation of type III interferon production	IL32
GO:1903436	regulation of mitotic cytokinetic process	NUP62
GO:1903438	positive regulation of mitotic cytokinetic process	NUP62
GO:1903490	positive regulation of mitotic cytokinesis	NUP62
GO:1904161	DNA synthesis involved in UV-damage excision repair	POLD3
GO:0032954	regulation of cytokinetic process	NUP62
GO:0045163	clustering of voltage-gated potassium channels	KCNIP2
GO:0098912	membrane depolarization during atrial cardiac muscle cell action potential	CACNA1C
GO:1900623	regulation of monocyte aggregation	CD44
GO:1900625	positive regulation of monocyte aggregation	CD44
GO:1902304	positive regulation of potassium ion export	KCNIP2
GO:1902499	positive regulation of protein autoubiquitination	MTA1
GO:1903766	positive regulation of potassium ion export across plasma membrane	KCNIP2
GO:0086001	cardiac muscle cell action potential	KCNIP2; CACNA1C
GO:0034343	type III interferon production	IL32
GO:0034344	regulation of type III interferon production	IL32
GO:0035585	calcium-mediated signaling using extracellular calcium source	CACNA1C
GO:1905636	positive regulation of RNA polymerase II regulatory region sequence-specific DNA binding	IL32
