# Supplementary material for: The Association between the Differential Expression of lncRNA and Type 2 Diabetes Mellitus in People with Hypertriglyceridemia
Source: Int J Mol Sci. 2023 Feb 21;24(5):4279. doi: 10.3390/ijms24054279 (PMC10002095; doi:10.3390/ijms24054279)
Supplement: Supplementary file 1 [file ijms-24-04279-s001.zip › Table S6.pdf]

Table S6 GO enrichment analysis of mRNAs in ceRNA networks (top 20 pathways of CC)

GO ID	Term	Input
GO:0005925	focal adhesion	CD44;RPL5;MPRIP;CSRP1;ANXA6
GO:0005924	cell-substrate adherens junction	CD44;RPL5;MPRIP;CSRP1;ANXA6
GO:0030055	cell-substrate junction	CD44;RPL5;MPRIP;CSRP1;ANXA6
GO:0005737	cytoplasm	IL32;CD44;TDP1;TRIT1;POLD3;DPYSL2;PHACTR2;DPH5;KCNIP2;RPL5;REEP5;MPRIP;AOPEP;MTG1;TMEM25;ABCB9;CACNA1C;CSRP1;RCAN1;ICOSLG;DNHD1;CRIP2;MTA1;ANXA6;NUP62;HOXB7
GO:0005912	adherens junction	CD44;RPL5;MPRIP;CSRP1;ANXA6
GO:0070161	anchoring junction	CD44;RPL5;MPRIP;CSRP1;ANXA6
GO:0035692	macrophage migration inhibitory factor receptor complex	CD44
GO:0016035	zeta DNA polymerase complex	POLD3
GO:0036156	inner dynein arm	DNHD1
GO:0043625	delta DNA polymerase complex	POLD3
GO:0005642	annulate lamellae	NUP62
GO:0043232	intracellular non-membrane-bounded organelle	POLD3;DPYSL2;RPL5;MPRIP;AOPEP;MTG1;CACNA1C;CSRP1;ICOSLG;NSMCE1;DNHD1;MTA1;NUP62;HOXB7
GO:0043228	non-membrane-bounded organelle	POLD3;DPYSL2;RPL5;MPRIP;AOPEP;MTG1;CACNA1C;CSRP1;ICOSLG;NSMCE1;DNHD1;MTA1;NUP62;HOXB7
GO:0030915	Smc5-Smc6 complex	NSMCE1
GO:0044613	nuclear pore central transport channel	NUP62
GO:0106068	SUMO ligase complex	NSMCE1
GO:0042824	MHC class I peptide loading complex	ABCB9
GO:0044424	intracellular part	IL32;CD44;TDP1;TRIT1;POLD3;DPYSL2;PHACTR2;DPH5;KCNIP2;RPL5;REEP5;MPRIP;AOPEP;MTG1;TMEM25;ABCB9;CACNA1C;CSRP1;RCAN1;ICOSLG;NSMCE1;DNHD1;CRIP2;MTA1;ANXA6;NUP62;HOXB7

GO:0005622	intracellular	IL32;CD44;TDP1;TRIT1;POLD3;DPYSL2;PHACTR2;DPH5;KCNIP2;RPL5;REEP5;MPRIP;AOPEP;MTG1;TMEM25;ABCB9;CACNA1C;CSRP1;RCAN1;ICOSLG;NSMCE1;DNHD1;CRIP2;MTA1;ANXA6;NUP62;HOXB7
GO:0030054	cell junction	CD44;RPL5;MPRIP;CACNA1C;CSRP1;ANXA6
