# Supplementary material for: The Association between the Differential Expression of lncRNA and Type 2 Diabetes Mellitus in People with Hypertriglyceridemia
Source: Int J Mol Sci. 2023 Feb 21;24(5):4279. doi: 10.3390/ijms24054279 (PMC10002095; doi:10.3390/ijms24054279)
Supplement: Supplementary file 1 [file ijms-24-04279-s001.zip › Table S7.pdf]

Table S7 GO enrichment analysis of mRNAs in ceRNA networks (top 20 pathways of MF)

GO ID	Term	Input
GO:0008092	cytoskeletal protein binding	DPYSL2;PHACTR2;MPRIP;CACNA1C;CSRP1;ANXA6;NUP62
GO:0004164	diphthine synthase activity	DPH5
GO:0017005	3'-tyrosyl-DNA phosphodiesterase activity	TDP1
GO:0052381	tRNA dimethylallyltransferase activity	TRIT1
GO:0042805	actinin binding	CACNA1C;CSRP1
GO:0004157	dihydropyrimidinase activity	DPYSL2
GO:0070259	tyrosyl-DNA phosphodiesterase activity	TDP1
GO:0051425	PTB domain binding	NUP62
GO:0086056	voltage-gated calcium channel activity involved in AV node cell action potential	CACNA1C
GO:0005250	A-type (transient outward) potassium channel activity	KCNIP2
GO:0015433	peptide antigen-transporting ATPase activity	ABCB9
GO:0015440	peptide-transporting ATPase activity	ABCB9
GO:0033220	amide-transporting ATPase activity	ABCB9
GO:0046923	ER retention sequence binding	KCNIP2
GO:0086007	voltage-gated calcium channel activity involved in cardiac muscle cell action potential	CACNA1C
GO:0019888	protein phosphatase regulator activity	PHACTR2;RCAN1
GO:0008270	zinc ion binding	TRIT1;AOPEP;CSRP1;CRIP2;MTA1
GO:0008597	calcium-dependent protein serine/threonine phosphatase regulator activity	RCAN1
GO:0046978	TAP1 binding	ABCB9
GO:0016812	hydrolase activity, acting on carbon-nitrogen (but not peptide) bonds, in cyclic amides	DPYSL2
