# Supplementary material for: The Association between the Differential Expression of lncRNA and Type 2 Diabetes Mellitus in People with Hypertriglyceridemia
Source: Int J Mol Sci. 2023 Feb 21;24(5):4279. doi: 10.3390/ijms24054279 (PMC10002095; doi:10.3390/ijms24054279)
Supplement: Supplementary file 1 [file ijms-24-04279-s001.zip › Table S8.pdf]

Table S8 KEGG enrichment analysis of mRNAs in ceRNA networks (top 20 pathways)

ID	Term	Input
ko04921	Oxytocin signaling pathway	RCAN1|CACNA1C
ko00730	Thiamine metabolism	NTPCR
ko03430	Mismatch repair	POLD3
ko03410	Base excision repair	POLD3
ko03030	DNA replication	POLD3
ko02010	ABC transporters	ABCB9
ko03420	Nucleotide excision repair	POLD3
ko03440	Homologous recombination	POLD3
ko04930	Type II diabetes mellitus	CACNA1C
ko04927	Cortisol synthesis and secretion	CACNA1C
ko04929	GnRH secretion	CACNA1C
ko04720	Long-term potentiation	CACNA1C
ko05031	Amphetamine addiction	CACNA1C
ko04924	Renin secretion	CACNA1C
ko05412	Arrhythmogenic right ventricular cardiomyopathy (ARVC)	CACNA1C
ko04742	Taste transduction	CACNA1C
ko04260	Cardiac muscle contraction	CACNA1C
ko04512	ECM-receptor interaction	CD44
ko04911	Insulin secretion	CACNA1C
ko04727	GABAergic synapse	CACNA1C
