# Supplementary material for: The Association between the Differential Expression of lncRNA and Type 2 Diabetes Mellitus in People with Hypertriglyceridemia
Source: Int J Mol Sci. 2023 Feb 21;24(5):4279. doi: 10.3390/ijms24054279 (PMC10002095; doi:10.3390/ijms24054279)
Supplement: Supplementary file 1 [file ijms-24-04279-s001.zip › Table S9.pdf]

Table S9 Details of type 2 diabetes mellitus pathway enrichment gene set

Symbol	Rank Metric Score	Running ES	Core enrichment
IRS1	1.294304848	0.084836	Yes
SOCS3	1.041809559	0.150220	Yes
SOCS1	0.778799415	0.170847	Yes
PIK3CD	0.720218122	0.197494	Yes
PIK3R3	0.620445549	0.178217	Yes
IRS2	0.597358286	0.200685	Yes
PKM	0.562897801	0.209039	Yes
MAPK3	0.535008013	0.218930	Yes
CACNA1C	0.533931375	0.252985	Yes
SOCS2	0.496268630	0.242655	Yes
MAPK9	0.479697585	0.255464	Yes
IKBKB	0.475783110	0.282480	Yes
PRKCE	0.428210229	0.247736	Yes
MAPK10	0.422711909	0.267422	Yes
TNF	0.389138192	0.244998	Yes
MTOR	0.384511143	0.264100	Yes
HK2	0.377907813	0.279634	Yes
PIK3CA	0.362728179	0.282266	Yes
KCNJ11	0.354062051	0.293279	Yes
PIK3R5	0.298998028	0.229953	No
PRKCD	0.295882493	0.244446	No
INSR	0.274092197	0.232575	No
PRKCZ	0.272141010	0.247627	No
PIK3R1	0.264947534	0.254314	No
MAPK1	0.226307184	0.213608	No
HK3	0.205583438	0.199649	No
SOCS4	0.200286895	0.205880	No
MAFA	0.192980751	0.210250	No
SLC2A4	0.177145228	0.203616	No
CACNA1A	0.163124815	0.198998	No
PIK3CG	0.142986119	0.189165	No
CACNA1B	0.085511476	0.140151	No
PIK3CB	0.056738634	0.120608	No
HK1	0.045205958	0.113891	No
ADIPOQ	0.007303623	0.071103	No
PIK3R2	-0.05313278	0.018758	No
GCK	-0.099194296	0.002398	No
CACNA1E	-0.143287912	-0.003720	No
MAPK8	-0.179446667	-0.002750	No
PKLR	-0.283411831	-0.008560	No
CACNA1D	-0.59910053	0.004947	No
