# Supplementary figures and images for: The Association between the Differential Expression of lncRNA and Type 2 Diabetes Mellitus in People with Hypertriglyceridemia
Source: Int J Mol Sci. 2023 Feb 21;24(5):4279. doi: 10.3390/ijms24054279 (PMC10002095; doi:10.3390/ijms24054279)

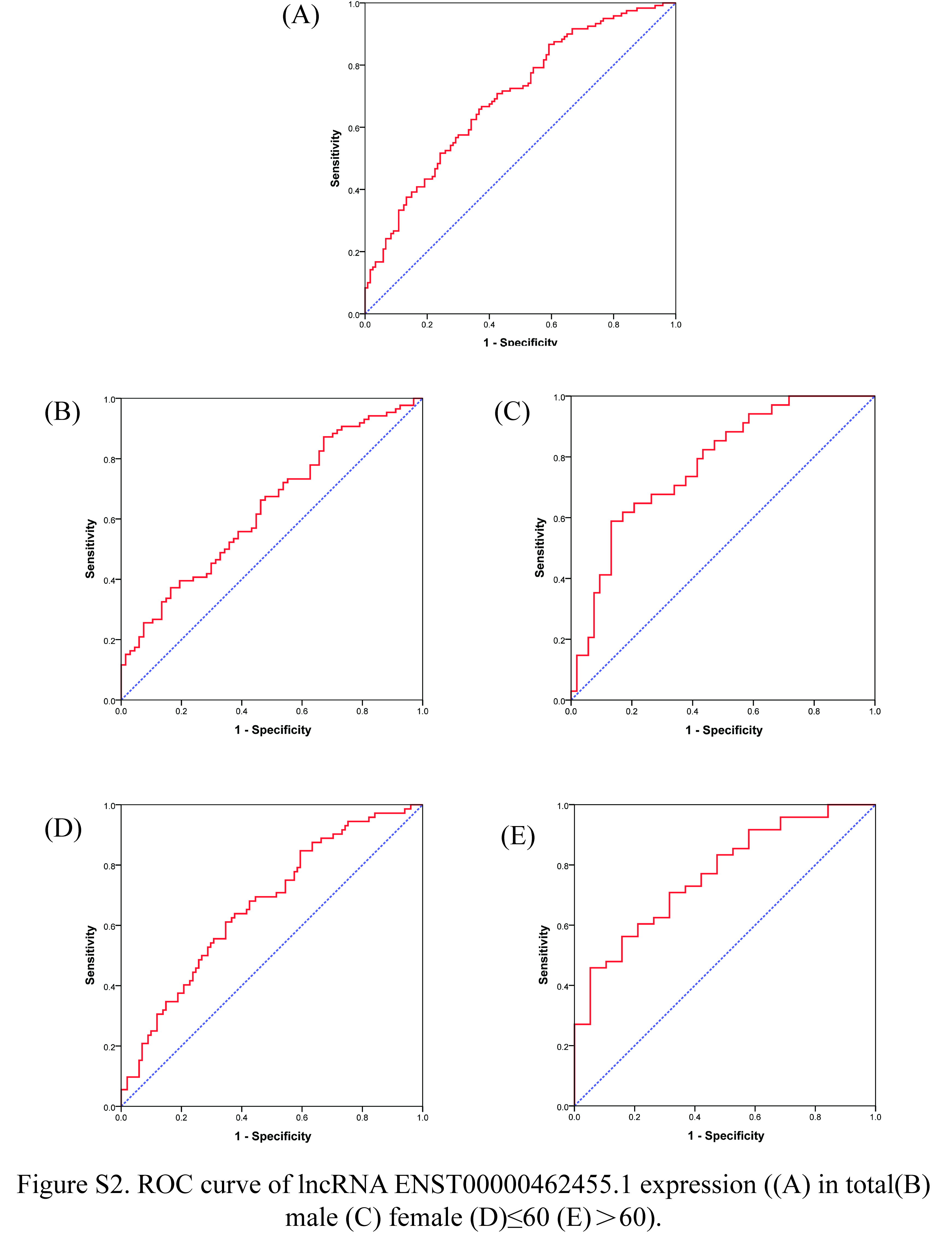

Supplement: Supplementary file 1 [file ijms-24-04279-s001.zip › Figure S2.tif]

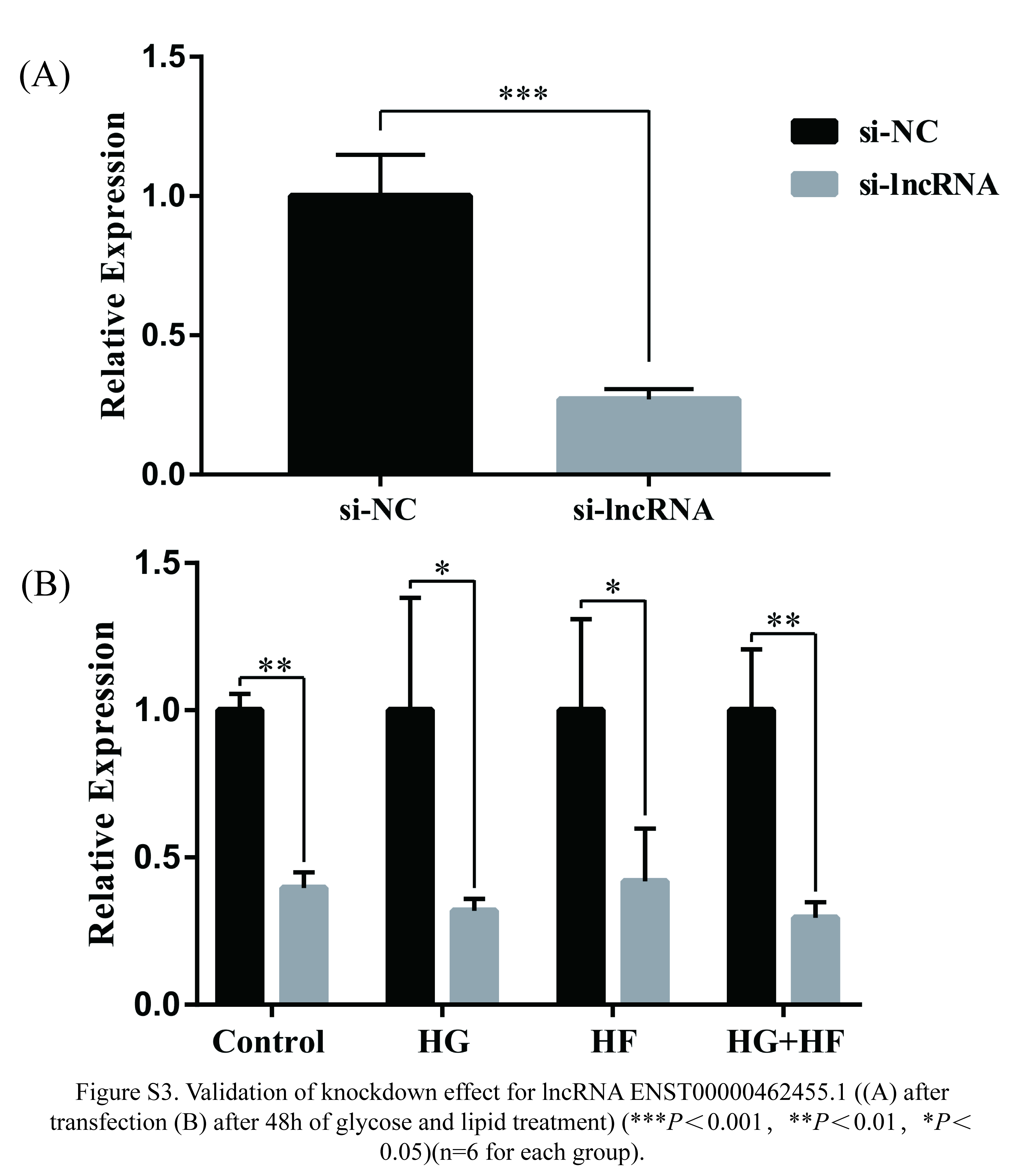

Supplement: Supplementary file 1 [file ijms-24-04279-s001.zip › Figure S3.tif]

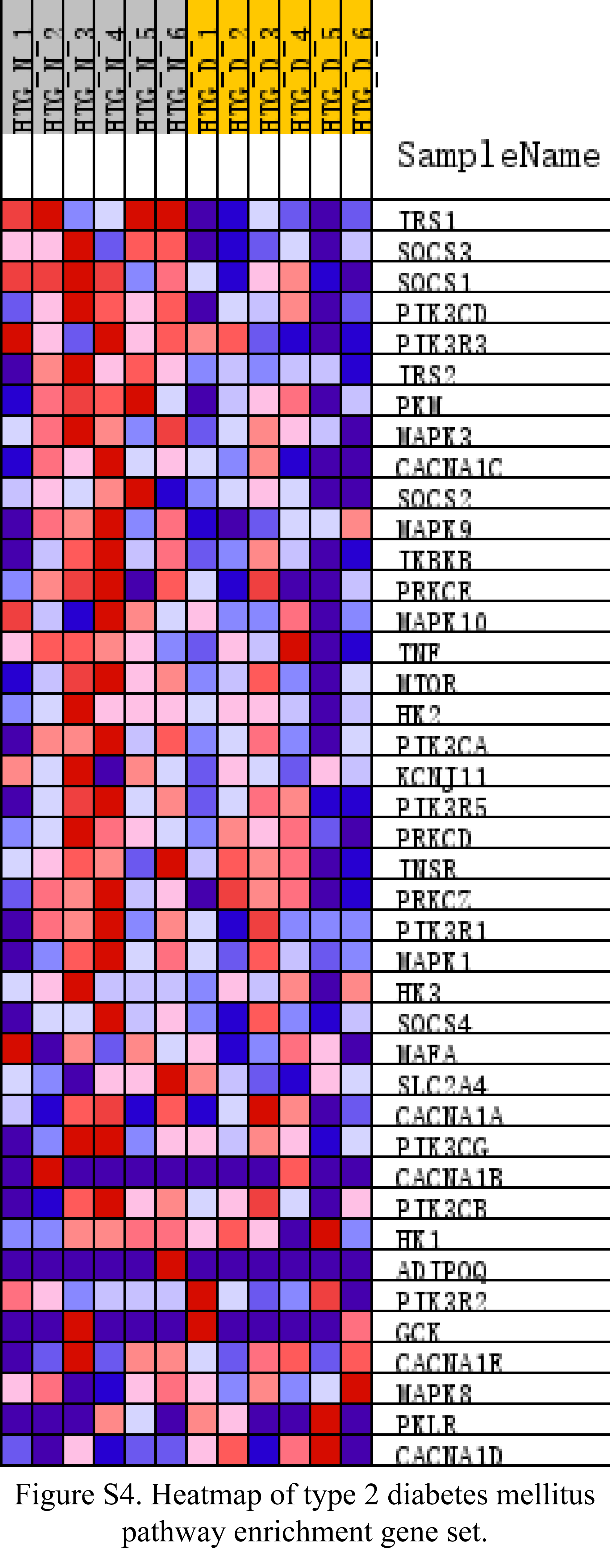

Supplement: Supplementary file 1 [file ijms-24-04279-s001.zip › Figure S4.tif]

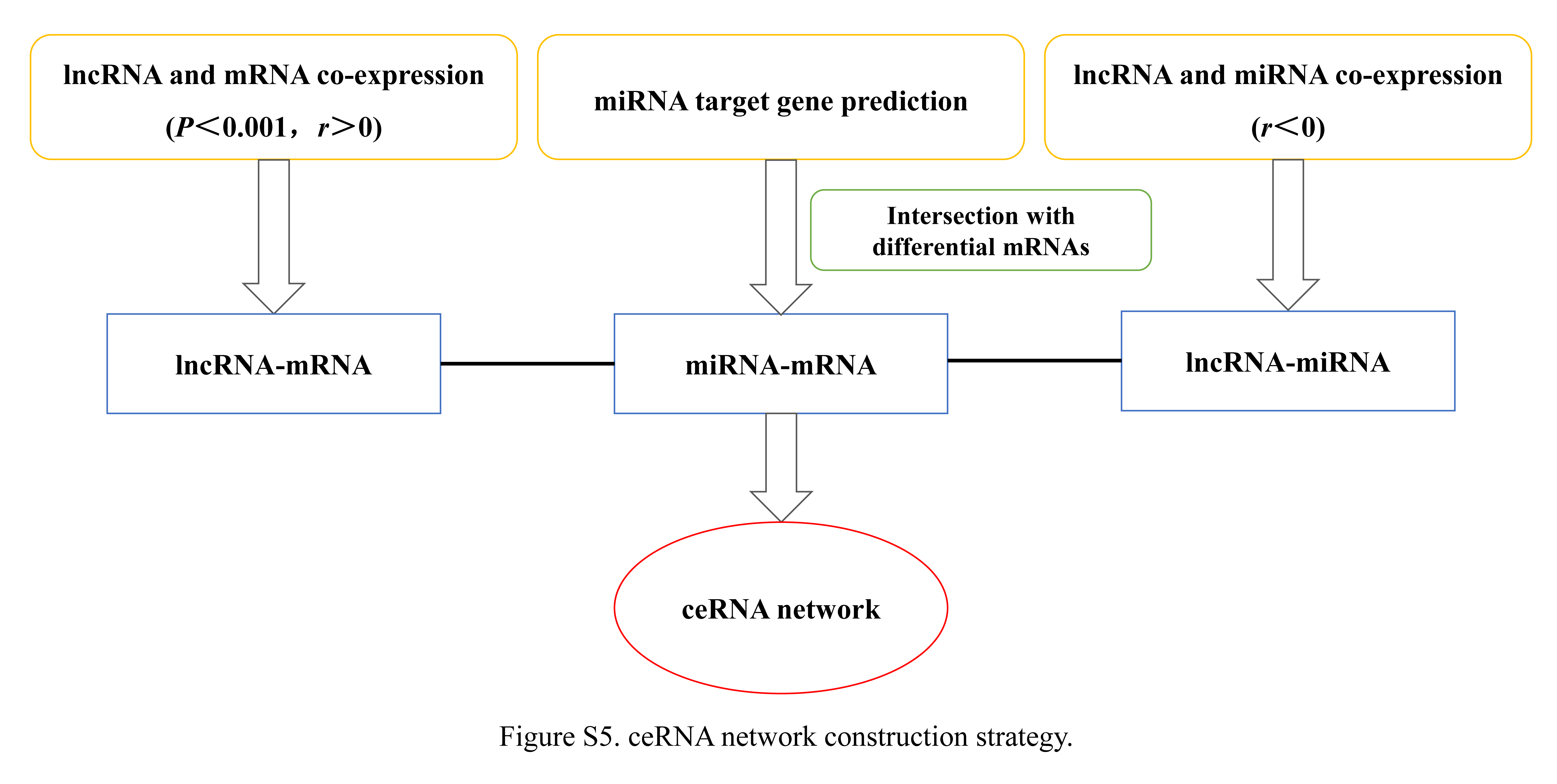

Supplement: Supplementary file 1 [file ijms-24-04279-s001.zip › Figure S5.tif]
